# The Future of Large Old Trees in Urban Landscapes

**DOI:** 10.1371/journal.pone.0099403

**Published:** 2014-06-18

**Authors:** Darren S. Le Roux, Karen Ikin, David B. Lindenmayer, Adrian D. Manning, Philip Gibbons

**Affiliations:** The Fenner School of Environment and Society, the Australian National University, Canberra, Australia; National University of Singapore, Singapore

## Abstract

Large old trees are disproportionate providers of structural elements (e.g. hollows, coarse woody debris), which are crucial habitat resources for many species. The decline of large old trees in modified landscapes is of global conservation concern. Once large old trees are removed, they are difficult to replace in the short term due to typically prolonged time periods needed for trees to mature (i.e. centuries). Few studies have investigated the decline of large old trees in urban landscapes. Using a simulation model, we predicted the future availability of native hollow-bearing trees (a surrogate for large old trees) in an expanding city in southeastern Australia. In urban greenspace, we predicted that the number of hollow-bearing trees is likely to decline by 87% over 300 years under existing management practices. Under a worst case scenario, hollow-bearing trees may be completely lost within 115 years. Conversely, we predicted that the number of hollow-bearing trees will likely remain stable in semi-natural nature reserves. Sensitivity analysis revealed that the number of hollow-bearing trees perpetuated in urban greenspace over the long term is most sensitive to the: (1) maximum standing life of trees; (2) number of regenerating seedlings ha^−1^; and (3) rate of hollow formation. We tested the efficacy of alternative urban management strategies and found that the only way to arrest the decline of large old trees requires a collective management strategy that ensures: (1) trees remain standing for at least 40% longer than currently tolerated lifespans; (2) the number of seedlings established is increased by at least 60%; and (3) the formation of habitat structures provided by large old trees is accelerated by at least 30% (e.g. artificial structures) to compensate for short term deficits in habitat resources. Immediate implementation of these recommendations is needed to avert long term risk to urban biodiversity.

## Introduction

Large old trees have been defined as keystone ecological structures because, relative to their size, they are disproportionate providers of resources crucial to other species [1], [2]. As trees mature, they begin to form a set of unique physical attributes or structural elements, including large volumes of coarse woody debris and litter, peeling bark, dead branches and hollows [3], [4]. Habitat structures provided by large old trees take centuries to form and are typically not provided by younger trees [5]. For example, hollows in *Eucalyptus* typically begin to form in trees 120–220 years old [6]. Hollows alone provide critical nesting resources for a diverse range of taxa worldwide, including invertebrates [7], reptiles [8], birds [9], and mammals [10].

Once large old trees are removed, they can be extremely difficult to replace in the short term because of the prolonged time period needed for trees to mature. This time lag can have serious ecological and management implications, particularly in modified landscapes where the rate of large old tree removal exceeds the rate of tree replacement [11], [12]–[14]. Species that depend on large old trees for survival (e.g. hollow-dependent fauna) may face extinction in the short term without actions that reverse current patterns of tree decline [2].

Human activities such as land clearance, logging and livestock grazing are responsible for the decline of large old trees in a diverse range of ecosystems, including: conifer forests in Europe [15] and North America [16], tropical rainforest in South America [17], and agricultural land in Australia [18]. However, few studies have investigated the decline of large old trees in urban landscapes [19], [20]. This is a major concern given the unprecedented rate of global urbanisation, one of the most rapid and destructive forms of land-use change [21], [22]. Population growth and rising demand for urban living space invariably puts pressure on existing urban habitat that can be important for biodiversity [23], [24], [25]. However, a great deal of uncertainty remains about the future of habitat structures in urban landscapes, especially structures like large old trees that are known to limit some species [26], [27]. Large old trees are especially vulnerable to removal in urban landscapes worldwide due to the potential safety risks posed to the public and infrastructure from falling branches or trees [20], [28], [29]. Therefore, obtaining information about the future availability of large old trees in urban landscapes is of high priority, especially for practitioners who are challenged by balancing urban growth and maintaining critical habitat for biodiversity over the long term.

Although there are parallels between urban landscapes and other modified environments (e.g. agricultural land), the management of trees in human-dominated urban settings poses a suite of unique and complex challenges. The key interacting drivers of tree loss in the urban matrix include: (1) urban sprawl and in-fill practices [30], (2) public safety policies that facilitate managed tree removal in existing greenspace to protect people and infrastructure [20], and (3) reduced tree regeneration [31]. Despite these challenges, urban environments also provide opportunities for innovative tree management, community engagement, people-led conservation strategies, and biodiversity offsets, which may include public tree planting initiatives and artificial nest box projects [32].

In this study, we used a simulation model to predict the future availability of native hollow-bearing trees in a rapidly expanding urban landscape. We used hollow-bearing trees as a surrogate for large old trees and other associated habitat structures such as coarse woody debris [4], [33], [34]. This is because it is well established that as trees age and their size increases so too does the probability of hollow occurrence [5], [35], [36]. Our four main study objectives were to: (1) compare future trajectories in hollow-bearing trees in urban greenspace with semi-natural nature reserves under existing land management practices; (2) identify which variables can be manipulated to increase the number of hollow-bearing trees occurring in urban greenspace over the long term; (3) test the efficacy of multiple alternative tree management strategies aimed at mitigating the decline of hollow-bearing trees; and (4) formulate recommendations that can be widely applied by practitioners to better maintain and perpetuate large old trees and their associated habitat structures in urban landscapes. Given the widespread nature of this issue in urban landscapes, we anticipate that our findings will be relevant to urban practitioners globally.

## Materials and Methods

### 2.1. Ethics statement

This research was conducted under ethical approval (protocol number A2012/37; The Australian National University Ethics Committee). Vegetation surveys undertaken on nature reserves and public greenspace were approved by permit from the ACT Government, Territory and Municipal Services in compliance with the Nature Conservation Act 1980. Field studies did not involve endangered or protected species.

### 2.2. Study area

We conducted our study in and around the city of Canberra, Australian Capital Territory (ACT), southeastern Australia (35° 17′ 35. 64″ S; 149° 07′ 27. 36″ E). Canberra is Australia's eighth largest city covering an area of 810 km^2^. The city supports a population of 375,000 people, which is projected to double by 2056 [37]. Canberra is a highly planned city described as the “Bush Capital” because of the extensive suburban tree cover and 34 nature reserves flanking the urban boundary [38]. The city is situated in the ecologically diverse Southern Tablelands region west of the Great Dividing Range. Lowland box-gum *Eucalyptus* woodlands and grasslands once dominated the region [39]. Box-gum grassy woodlands are characterised by two dominant species, yellow box (*Eucalyptus melliodora*) and Blakely's red gum (*E. blakelyi*) that occur in association with other eucalypt species, including apple box (*E. bridgesiana*), red box (*E. polyanthemos*), red stringybark (*E. macrorhyncha*), and scribbly gum (*E. rossii*). Extensive land clearance for stock grazing and urban development has led to a near 95% decline in intact box-gum grassy woodlands, which is now listed as a critically endangered ecological community [40]. What vegetation remains exists in semi-natural nature reserves or as highly modified isolated remnant patches and scattered paddock and urban trees [41], [42].

### 2.3. Sampling design

We confined our sampling effort to a single vegetation type: the predicted pre-European (pre-1750) extent of box-gum grassy woodland. Within this vegetation type, we stratified sampling according to two dominant land-use types and five geographic zones, creating a total of 10 strata. Our land-use types were: (1) nature reserves, which are designated semi-natural areas managed for conservation; and (2) urban greenspace, made up of publicly accessible parklands (60%), roadside margins (24%), remnant vegetation (9%), and sports grounds (7%). Urban greenspace accounted for 11% of the total urban environment in our study area. We divided our study landscape into five geographic zones to capture variability and avoid biasing sampling effort to areas with specific local or historical attributes (e.g. fire history). An equal number of fixed area plots (50×20 m; 0.1 ha) were randomly allocated by land-use type (n = 100) and geographic zone (n = 40). This resulted in a total of 200 plots or 20 ha of sampled land from 28 reserves and 100 urban greenspaces. Plots were >250 m apart to minimise spatial dependence and allocated to greenspace ≥0.2 ha.

### 2.4. Data collection

We measured the diameter at breast height over bark (DBH; 1.3 m above ground) of every living and dead tree in each plot. We measured only the largest stem of multi-stemmed trees [43]. Trees with stems <1.3 m above the ground were measured at the base of the stem. The number of naturally regenerating and planted seedlings ≤10 cm (DBH) were counted in each plot and formed the first size class of our tree population. We identified all living trees to species level. Each tree was inspected for hollows from all angles on the ground using binoculars (10×25). One observer (DSL) completed this task to reduce multi-observer bias and maintain consistency in hollow identification [44]. Our objective was not to determine the absolute number of hollows but rather relative hollow occurrence per tree. We selected a minimum entrance size of 2 cm for hollows. This was because: (1) the full range of hollow-dependent vertebrate taxa, including marsupials, birds, and bats, would be accounted for; and (2) hollows smaller than 2 cm were difficult to reliably identify from the ground [45].

### 2.5. Simulation model

The simulation model described in [12], tracks the mean DBH of trees, including hollow-bearing trees, in separate size cohorts over time. The model has pre-defined rates of tree mortality and recruitment applied at each time step. For this study, we ran separate simulations for native tree populations occurring in nature reserves and urban greenspace. Exotic trees were recorded only in the urban greenspace and accounted for 30% of all recorded trees. We excluded exotic trees from our analyses because only native trees were recorded with hollows in our study area. Simulation models for both land-use types were parameterised with the following baseline data: the current number of native trees in existing stands sorted by DBH cohort; the predicted age and growth rate of trees; the frequency of regeneration events; the number of seedlings at each regeneration event; and the rate of tree mortality.

**Figure 1:**
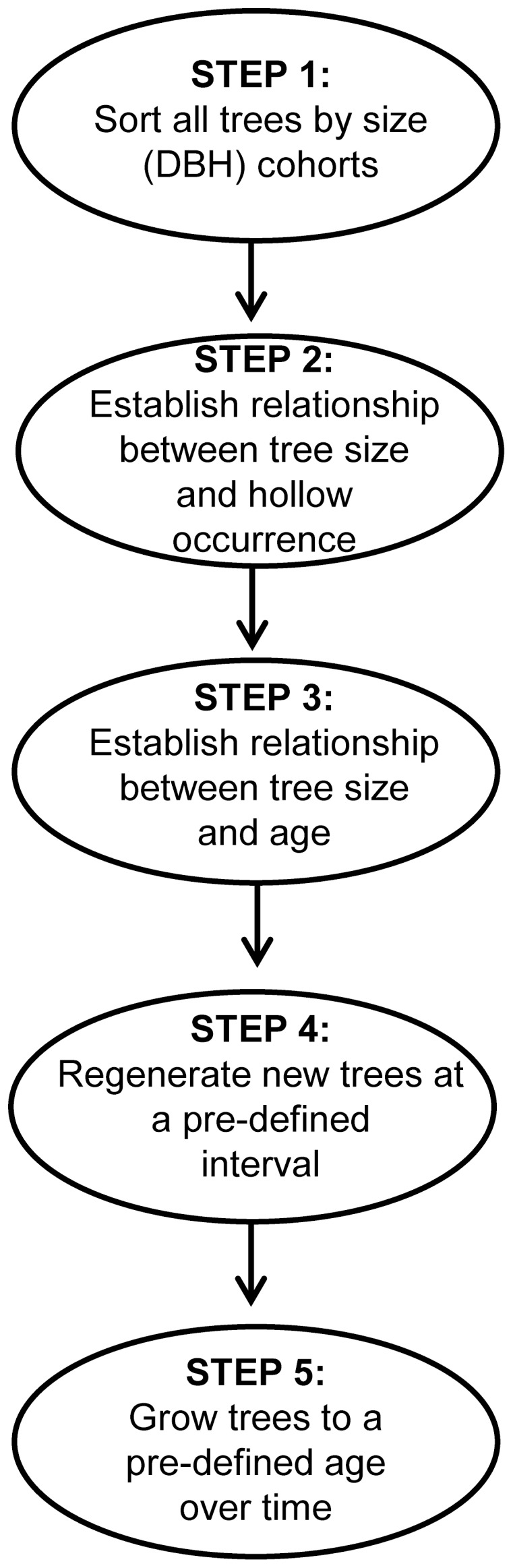
Simple schematic highlighting the five principle steps of our simulation model.

There were five principle steps in our modelling process (summarised in Fig. 1 and described further in Summary S1):

(1) We calculated the mean number of trees in 10 cm DBH size cohorts (ranging from 0.1–10 cm to >100 cm) for each native tree species and dead trees, using data collected in each land-use type (Table S1).

(2) We used a generalised logistic regression model with a binomial distribution and logit link to establish a relationship between hollow occurrence (i.e. the presence of at least one hollow ≥2 cm; binary response) and tree size (i.e. DBH; explanatory variable). We also fitted tree species as an explanatory variable in our model. Based on correlations in hollow occurrence by DBH between individual species, we identified three distinct species groupings. Species group one included yellow box, apple box, brittle gum (*E. mannifera*), broad-leaved peppermint (*E. dives*), bundy (*E. goniocalyx*), mealy bundy (*E. nortonii*), brown barrel (*E. fastigata*), alpine ash (*E. delegatensis*), ribbon gum (*E. viminalis*), mountain gum (*E. dalrympleana*), candlebark (*E. rubida*) and ironbark (*E. sideroxylon*). Group two included Blakely's red gum, red box, red stringybark and scribbly gum. Group three was dead trees. We found that species groups differed significantly (Wald statistic  = 101.5; *P*<0.001) from each other (Table 1). The relationship between tree size and hollow presence was highly significant in our model (Wald statistic  = 388.1; *P*<0.001). The area under the receiver operating characteristic curve of our model was 0.92, indicating that the discriminating ability of our model was excellent [46]. For each species group, we derived separate model equations which took the form: *Logit (Pr. Hollows)* = −7.112+(0.086 x DBH) + (species group estimate).

**Table 1:** Generalised logistic regression model used to predict the proportion of hollow-bearing trees in each 10 cm DBH (diameter at breast height) cohort.

Variables	Coefficient	Standard error	Lower 95% confidence interval	Upper 95% confidence interval	*P*-value
Intercept	−7.112	0.335	−7.769	−6.456	<0.001***
Species group 1	0.000	-	-	-	-
Species group 2	1.413	0.274	0.876	1.949	<0.001***
Species group 3	3.861	0.383	3.110	4.613	<0.001***
DBH	0.086	0.004	0.077	0.095	<0.001***

Coefficients, standard errors, 95% confidence intervals, and *P*-values are presented with species group one held as the reference level.

(3) We established a relationship between DBH and tree age using the following equation: Age  = 0.02×π×(DBH _standardised_/2)^2^, where DBH _standardised_ is the yellow box equivalent diameter for each tree. Yellow box is the only tree species for which data exist to establish a relationship between age and DBH [47]. We scaled all DBH values for each tree species relative to that of a yellow box equivalent using the method described in [18], [26]. To do this, we first calculated each DBH value as a proportion of the maximum DBH recorded for each tree species and then multiplied this value by the largest DBH recorded for yellow box in our study area (151 cm). Therefore, we assumed that all species had proportionally equal growth rates that were similar to that of yellow box. Although this approach is not ideal because it is unlikely to yield precise age estimates for each species, it currently is the most practicable solution available in the absence of age-DBH relationship data for other eucalypt species [26], [48]. Therefore, our model had a degree of uncertainty related to tree growth rates, as these data likely differ for each species. However, a previous study [12] found that long-term predictions for mature trees is not sensitive to uncertainty in this variable and suggests that the focus should instead be on testing the effects of uncertainty for other parameters in the model.

(4) We simulated tree regeneration in both land-use types to ensure that uncertainties associated with regeneration were reflected in our models. Tree regeneration is an event-driven process that can be sporadic and influenced by natural phenomena and/or anthropogenic factors such as climate, competition, and planting effort [31], [49]. At each regeneration event, viable seedlings may or may not establish and survive over time. To simulate these uncertainties, the number of seedlings ha^−1^ for each run of our model was drawn randomly from a Poisson distribution with the mean equal to the mean number of trees recorded in the 0–10 cm DBH cohort for each species group. For species group one and two in urban greenspace, the mean number of trees in the 0–10 cm DBH cohort was 11 and 13 seedlings ha^−1^, respectively. For species group one and two in nature reserves, the mean number of trees in the 0–10 cm DBH cohort was 119 and 193 seedlings ha^−1^, respectively. The time-step for each run of the model was equivalent to the average age of trees in the 0–10 cm DBH cohort for both land-use types, which was approximately 8 years.

(5) Annual tree mortality was modelled in a density-dependent manner to reflect declines in the number of trees over successive DBH cohorts or as trees age. Therefore, we assumed that tree densities would naturally thin out over time due to factors such as competition among conspecifics [50]. To simulate this process, we calculated annual mortality for each DBH cohort using the equation: 1 - *s* ^(1/*y*)^, where *s* is the proportion of trees that survive from one cohort to the next, and *y* is the number of years it takes trees to progress from one cohort to the next by 10 cm DBH increments. However, in some urban greenspaces (e.g. roadside margins), density-dependent mortality may be less pronounced as tree survivability may instead be predominantly influenced by tree planting and protection efforts. Therefore, for urban greenspace, we also tested the mean annual mortality rate across all cohorts, which yielded similar model trajectories to density-dependent mortality. We decided to apply density-dependent mortality to both land-use types for consistency and because a majority of urban greenspace sampled constituted parklands and remnant vegetation where natural regeneration and density-dependent mortality may still occur. We set 500 years as the maximum age that living trees will remain standing in both land-use types. This is based on the only longevity estimate available for eucalypts in our study area [47]. It is reasonable to assume that for other eucalypt species this age would also be the upper limit of survivability. Therefore, model uncertainties pertaining to species longevity are likely to be over-estimated and based on a best-case longevity. We assumed that once trees died in urban greenspace, they no longer functioned as hollow-bearing trees into the next time step. This is based on local tree management policies that facilitate dead tree removal on public land [51]. However, for nature reserves, we conservatively estimated that dead trees could remain standing for at least 50 years after initial mortality (i.e. 550 years in total), based on observations of the standing life of dead trees in *Eucalyptus* forests [52], however, we acknowledge the paucity of available data to support this estimate.

### 2.6. The availability of hollow-bearing trees under existing management practices

We used our simulation model, parameterised with those data detailed above, to predict the mean number of hollow-bearing trees ha^−1^ occurring in nature reserves and urban greenspace over time under existing land management protocols. Simulations were undertaken over 300 years using a Monte Carlo simulation based on 300 runs of our model (i.e. the number of iterations required for relatively well-defined distributions). This approach relies on random sampling over multiple simulations to generate probabilities in a heuristic manner [53]. Therefore, for each run of our model, input data for several variables were drawn randomly from defined distributions. The number of recruits was drawn from a Poisson distribution (step 4 above). Annual mortality was drawn from a normal distribution, where negative values were converted to zero. The maximum standing life of living trees was held at 500 years for nature reserves. However, for urban greenspace, values were drawn from a uniform distribution between 60 years (the estimated minimum standing life of trees in our study area) [54] and 500 years (the estimated maximum standing life of trees in our study area). This range of lifespans reflects variation in current tree management practices in different types of urban greenspace. Variables held constant in our model were the period between regeneration events (8 years) and coefficients for the age-DBH (0.019) and DBH-hollow (1.413) relationships.

### 2.7. Variables that can be manipulated to mitigate the decline of hollow-bearing trees

We performed a sensitivity analysis, as described in [55], to identify which variables can be manipulated in urban greenspace to mitigate the decline of hollow-bearing trees. For this analysis, we also used a Monte Carlo simulation based on 300 runs of our model. We repeatedly populated each run of the model with data drawn randomly from uniform distributions for each variable. Where applicable, values were drawn from a wider range than observed under existing management practices to more broadly test a range of alternative management strategies. Variables that can be manipulated by management included: (1) maximum standing life of trees (range: 60–500 years for species groups one and two, based on longevity estimates for urban trees in our study area); (2) number of seedlings ha^−1^ (range: 0–60 seedlings ha^−1^ for species groups one and two, testing various regeneration targets); (3) period between regeneration events (range: 1–50 years, testing various regeneration schedules); (4) rate of annual mortality (range: 0.03–0.1 model coefficients, testing various feasible survivability outcomes); and (5) rate of hollow formation (range: 1.5–3.7 model coefficients, testing a range of hollow acceleration strategies above an observed existing rate (i.e. 1.4) up to a rate observed for dead trees (i.e. 3.8) in our study area, which we assumed indicated a maximal hollow formation rate for living trees). We fixed the coefficient for the DBH-age relationship at 0.019 assuming that this could not be changed appreciably.

We used linear regression to test the relative sensitivity of our response variable (i.e. the mean number of hollow-bearing trees ha^−1^) against the explanatory variables that are the parameters in our simulation model. We natural log-transformed (ln (x+1)) our response to satisfy assumptions of normality. There were no significant interactions between explanatory variables and interaction terms were dropped from the final additive model. We used stepwise regression to determine the model of best fit. Percentage variance accounted for by our final model was 40%. Due to the high number of replications used in simulation models, it is inappropriate to rely on conventional *P*-values to indicate statistical significance [56]. Instead, we used relative effect size, as indicated by variance ratios, to identify the most sensitive parameters in our model. Variance ratios were calculated as the mean square of each term change divided by the residual mean squares of the original maximal model (Table 2). Predictions are presented only for variables with the greatest relative effect sizes (i.e. most ecologically important), where all other explanatory variables are held at their mean model values.

**Table 2:** Linear regression model used to perform a sensitivity analysis of the mean number of hollow-bearing trees ha^−1^ (ln (x+1) transformed) perpetuated in urban greenspace over 300 years.

Variables	Mean	Standard deviation	Coefficient	Standard error	Variance ratio
Intercept	-	-	0.602	0.204	-
Maximum standing life (years)	274.10	88.04	0.004	0.0003	138.61
Number of seedlings ha^−1^	31.03	12.09	0.009	0.002	13.81
Rate of hollow formation (coefficient)	2.59	0.17	0.151	0.042	11.04
Rate of annual mortality (coefficient)	0.06	0.02	−1.290	1.450	0.31
Period between regeneration (years)	24.74	13.40	0.000	0.002	0.00

Means, standard deviations, coefficients, standard errors, and variance ratios, which indicate the relative importance or effect size of each model term, are presented for each explanatory variable used to parameterise our simulation model.

### 2.8. The availability of hollow-bearing trees under alternative management strategies

We also simulated a series of alternative management strategies using our simulation model. We modelled the mean number of hollow-bearing trees ha^−1^ occurring in urban greenspace over 300 years. Scenarios were based on either: (1) a management strategy that manipulates only a single variable up to the maximum value defined in our regression model described above, or (2) a combined management strategy that manipulates all three variables for a set of values that we deemed most practicable for urban landscapes given other socio-economic constraints. Variables not manipulated were fixed at their mean values under existing management practices. In all simulated scenarios, management actions were assumed to take effect immediately. Statistical analyses were completed using GenStat (15^th^ edition, VSN International Ltd, Hemel Hempstead, UK).

## Results

**Figure 2:**
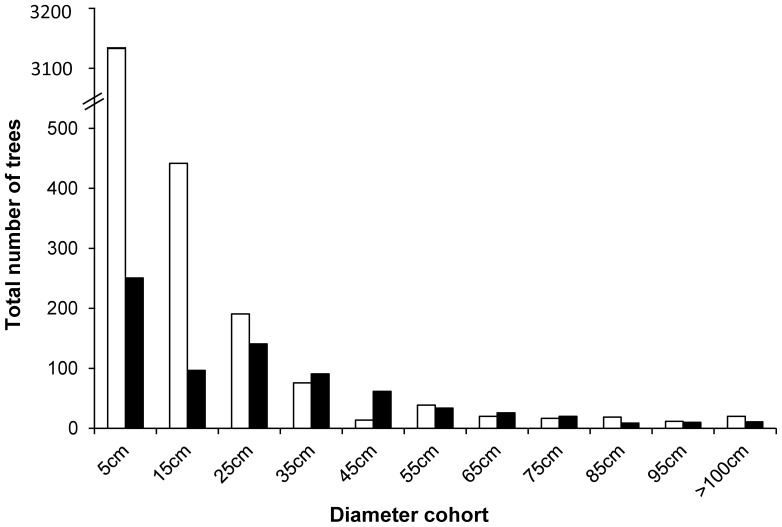
Frequency distribution of median tree diameter cohorts for tree stands (all species) in nature reserves (open bars) and urban greenspace (solid bars).

We recorded a total of 4,865 trees belonging to 16 eucalypt species. Of those trees, 85% (4,111 trees) were recorded in nature reserves and 15% (754 trees) in urban greenspace. The key difference between tree populations in nature reserves and urban greenspace was the number of seedlings recorded in the 0.1–10 cm DBH cohort (Fig. 2). In reserves, we recorded 315 seedlings ha^−1^, which was 13 times the number recorded in urban greenspace, with 25 seedlings ha^−1^.

### 3.1. The availability of hollow-bearing trees under existing management practices

**Figure 3:**
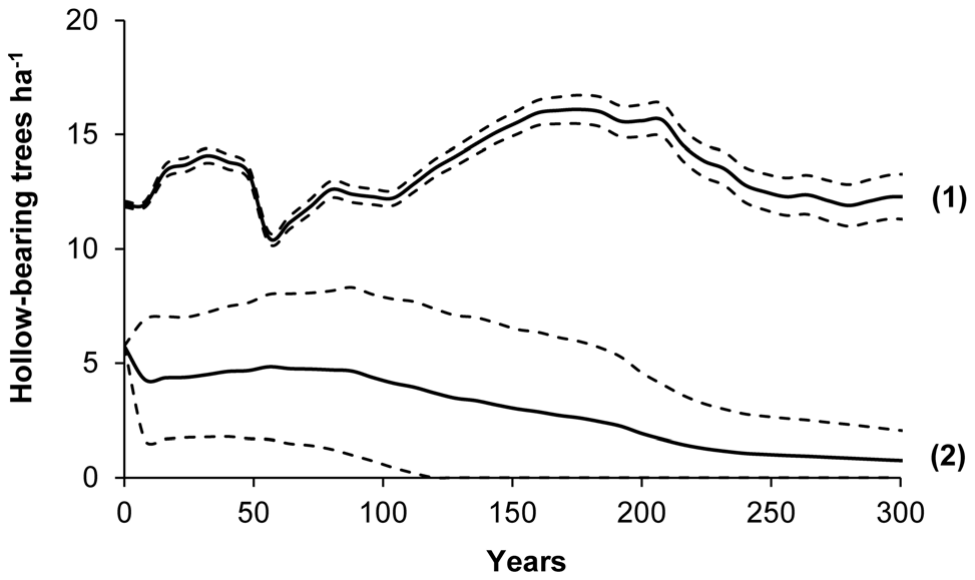
Simulations predicting the relative number of hollow-bearing trees ha^−1^ (mean ±95% prediction interval) over 300 years under existing management practices in nature reserves (1) and urban greenspace (2).

In urban greenspace, we found that under existing management practices, the mean number of hollow-bearing trees ha^−1^ is predicted to decline by 87% over 300 years from an initial recorded stand density of 5.74 trees ha^−1^ to 0.76 trees ha^−1^ (Fig. 3). Conversely, in nature reserves, hollow-bearing tree densities fluctuate around a relatively stable mean density of 13.4 trees ha^−1^. Prediction intervals for urban greenspace were more variable around the mean than for nature reserves. This is driven by highly variable standing lives that trees are permitted to reach in different urban greenspaces (i.e. 60–500 years old). Prediction intervals indicate that under a worst case scenario (i.e. lower 95% prediction interval) all hollow-bearing trees may be lost from urban greenspace within 115 years. Even under a best case scenario (i.e. upper 95% prediction interval) hollow-bearing trees steadily decline over time.

### 3.2. Variables that can be manipulated to mitigate the decline of hollow-bearing trees

Sensitivity analysis revealed that the mean number of hollow-bearing tree ha^−1^ was most sensitive to: (1) the maximum standing life of trees; (2) the number of seedlings ha^−1^; and (3) the rate of hollow formation (Table 2). The mean number of hollow-bearing trees ha^−1^ was least sensitive to the period between regeneration events and annual mortality. We also did not identify meaningful interactions between maximum standing life and annual mortality, maximum standing life and the rate of hollow formation, and the number of seedlings ha^−1^ and the period between regeneration events.

#### 3.2.1. Maximum standing life

**Figure 4:**
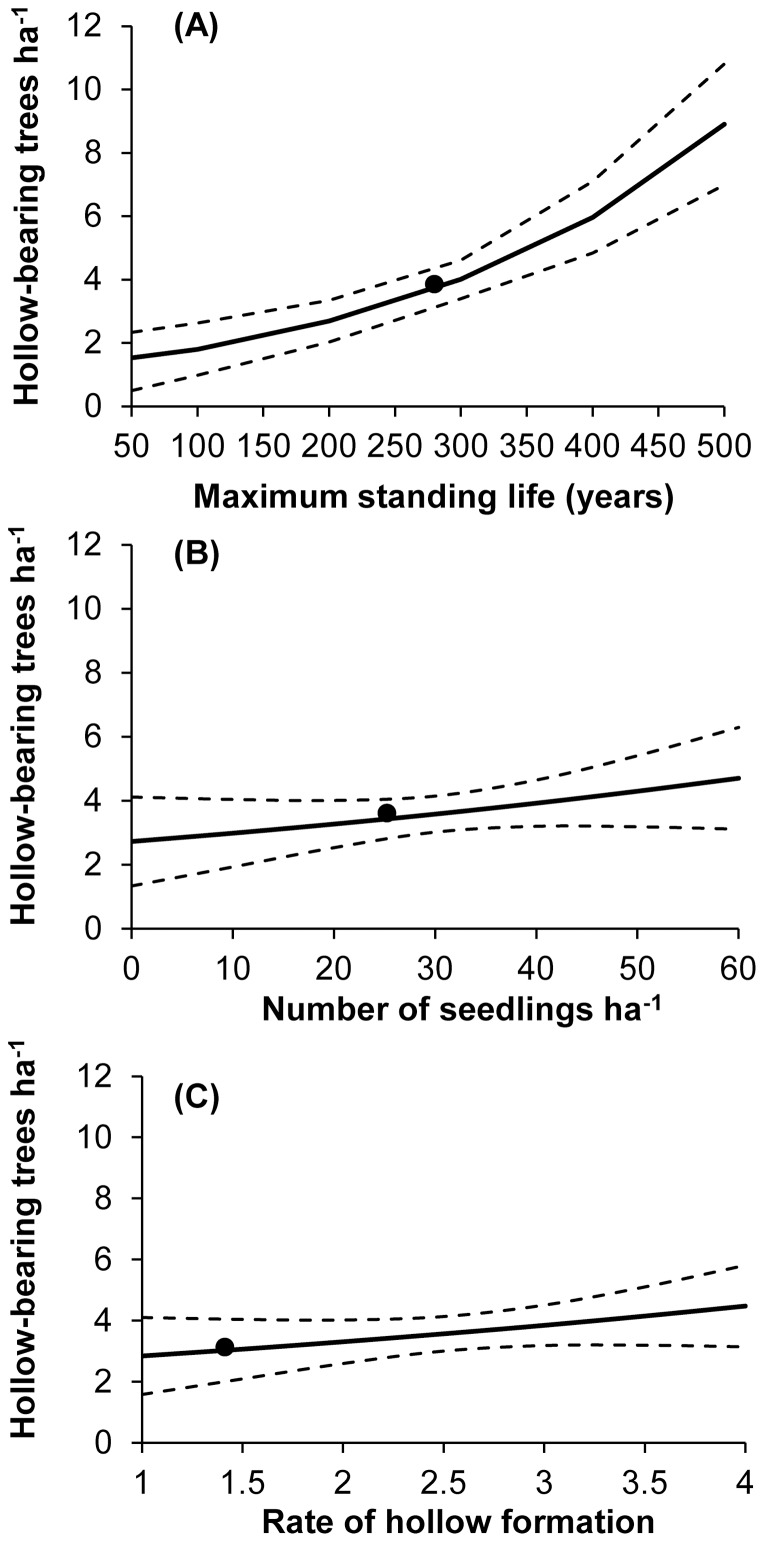
The predicted relative number of hollow-bearing trees ha^−1^ (mean ±95 prediction intervals) in urban greenspace over 300 years for a range of values for variables with the greatest relative effect sizes derived from a sensitivity analysis. Variables include: the maximum standing life of trees (A); the number of seedlings ha^−1^ (B); and the rate of hollow formation (represented by the coefficient for the probability of hollow occurrence; C). Predicted thresholds under existing management practices are provided for reference (solid circles).

The number of hollow-bearing trees perpetuated in urban greenspace over the long term was most sensitive to the maximum standing life of trees (variance ratio  = 138.61). We predicted that hollow-bearing trees would increase in urban greenspace by approximately 0.8 trees ha^−1^ (22%) for each additional 50 years that trees are permitted to remain standing (Fig. 4A).

#### 3.2.2. Number of seedlings

**Figure 5:**
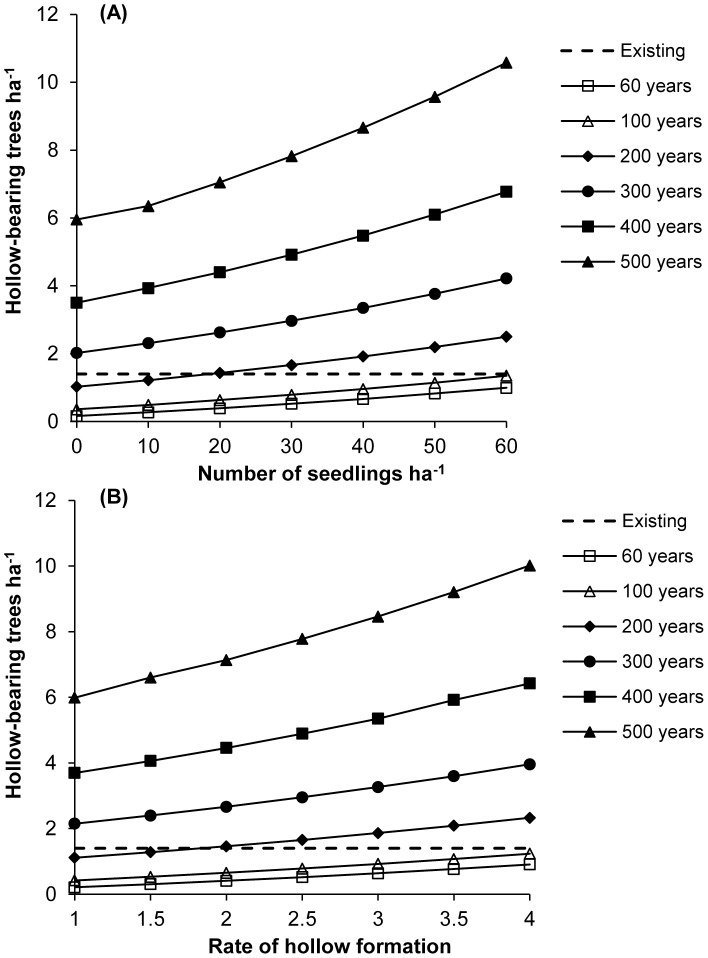
The predicted relative mean number of hollow-bearing trees ha^−1^ in urban greenspace over 300 years for a combination of values for: the maximum standing life of trees and the number of seedlings ha^−1^ (A); and the maximum standing life of trees and the rate of hollow formation (represented by the coefficient for the probability of hollow occurrence; B).

The number of seedlings ha^−1^ also contributed to the number of hollow-bearing trees perpetuated in urban greenspace over the long term, although relative to maximum standing life this contribution was smaller (variance ratio  = 13.81). We predicted that for every 10 additional native seedlings ha^−1^, the number of hollow-bearing trees would increase by 0.3 trees ha^−1^ (10%; Fig. 4B). However, we predicted that to perpetuate hollow-bearing trees even marginally above existing levels will require at least 30 seedlings ha^−1^ and all trees to remain standing for at least 200 years (Fig. 5A).

#### 3.2.3. Rate of hollow formation

Similarly, the rate of hollow formation also contributed to the number of hollow-bearing trees perpetuated in urban greenspace over the long term, although relative to maximum standing life this contribution was smaller (variance ratio  = 11.04). We predicted that hollow-bearing trees would increase by 0.2 trees ha^−1^ (8%) for every 0.5 increase in the rate of hollow formation (Fig. 4C). However, we predicted that to perpetuate hollow-bearing trees even marginally above existing levels will require accelerating hollow formation to a rate of 2.5 (i.e. a 44% increase above the observed mean rate) and all trees to remain standing for at least 200 years (Fig. 5B).

### 3.3. The availability of hollow-bearing trees under alternative management strategies

#### 3.3.1. Isolated management approach

**Figure 6:**
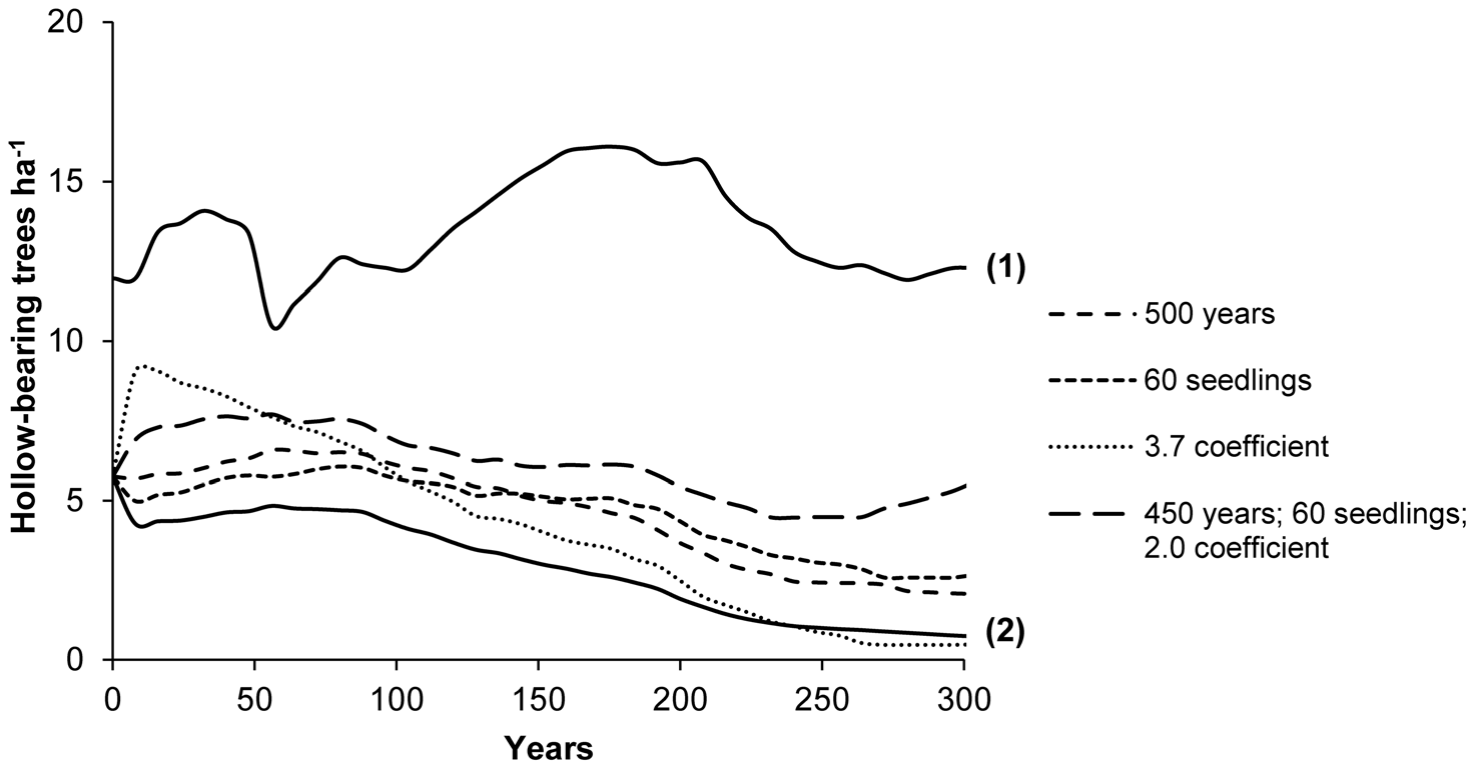
The predicted relative mean number of hollow-bearing trees ha^−1^ over 300 years under a series of alternative urban tree management scenarios (dashed lines). Simulated scenarios include: increasing the standing life of trees only up to 500 years; increasing the number of seedlings only up to 60 ha^−1^; accelerating hollow formation only by 62% above the observed mean rate (as represented by the coefficient for the probability of hollow occurrence); and a combined management approach (i.e. our recommended management proposal), which manipulates all three variables simultaneously. Scenarios under existing management practices are provided for reference by solid black lines for nature reserves (1) and urban greenspace (2).

If tree standing life were maximised to 500 years and all other variables were unchanged (i.e. held at their mean values under existing management practices), then the mean number of hollow-bearing trees in urban greenspace is predicted to still decline by 64% over the long term, from an initial stand density of 5.74 trees ha^−1^ to 2.09 trees ha^−1^ (Fig. 6). If the number of seedlings ha^−1^ were increased only to 60 seedlings ha^−1^, then the mean number of hollow-bearing trees in urban greenspace is predicted to still decline by 53% over the long term, from an initial stand density of 5.74 trees ha^−1^ to 2.68 trees ha^−1^. If hollow formation were accelerated only to a rate of 3.7 (i.e. the maximum rate of hollow formation observed for living trees and a 62% increase above the observed mean rate), then the mean number of hollow-bearing trees in urban greenspace is predicted to initially increase to 9 trees ha^−1^ in the short term, but decline by 92% to 0.46 tree ha^−1^ over the long term.

#### 3.3.2. Combined management approach

In contrast, a combined management approach that manipulates all sensitive explanatory variables is predicted to increase the number of hollow-bearing trees ha^−1^ over the long term (Fig. 6). To achieve this will require at least: (1) increasing the standing life of trees to 450 years (approximately 40% longer average lifespans); (2) increasing the number of seedlings to 60 seedlings ha^−1^ (approximately 60% greater regeneration rate); and accelerating hollow formation up to a rate of 2.0 (approximately 30% greater hollow formation rate; see Table 3). Under this scenario, the density of hollow-bearing trees will initially need to be actively increased in the short term by accelerating hollow formation to achieve at least 7 hollow-bearing trees ha^−1^. Over time, the density of hollow-bearing trees is predicted to first gradually decline before an increase occurs within 250 years.

**Table 3:** Summarised values for each variable used to parameterise our simulation model under existing management practices for nature reserves and urban greenspace.

Variables	Nature reserves	Urban greenspace	Urban management recommendations
Maximum standing life (years)	500	60–500	450 (∼40% increase)
Number of seedlings ha^−1^(all species)	315	25	60 (∼60% increase)
Rate of hollow formation	1.4	1.4	2.0 (∼30% increase)
Rate of annual mortality	0.03	0.06	-
Period between regeneration (years)	8	8	-

Relative values are derived from raw vegetation data or, where applicable, published estimates. Urban management recommendations, derived from a series of simulated alternative management strategies, are indicated for variables identified as being the most ecologically important from a sensitivity analysis.

## Discussion

Large old trees support unique habitat structures (e.g. hollows, coarse woody debris), which form over extensive time periods and cannot be provided by younger trees [5], [6]. The decline of large old trees in modified landscapes is a global conservation issue that has serious implications for biodiversity [11]. To date, few studies have addressed this problem in urban landscapes, which is a growing concern given the unprecedented rates of urbanisation in cities worldwide [25]. Using a simulation model, we investigated the decline of large old trees in an urban landscape over centuries. We predicted that hollow-bearing trees (a surrogate for large old trees) will decline by 87% over 300 years in urban greenspace under existing management practices. Under a worst case scenario, hollow-bearing trees may be entirely lost from urban greenspace within 115 years. Our analysis revealed that the decline of hollow-bearing trees in urban greenspace is most sensitive to: the maximum standing life of trees, the number of regenerating seedlings ha^−1^, and the rate of hollow formation. To mitigate the decline of large old trees in urban greenspace over the long term, we recommend a management strategy that collectively: (1) maximises the standing life of trees, (2) increases tree regeneration rates, and (3) accelerates the formation of habitat structures provided by large old trees. These results, and the methods used, have important implications for ecologically sustainable urban development.

### 4.1. Existing management practices

Our results provide further evidence that urban landscapes face a concerning future of large old tree decline, which is comparable with other highly impacted landscapes, including agricultural land [12], [57] and production forests [15], [52]. We argue that predicted declines in hollow-bearing trees in urban greenspace (Fig. 3) will not only negatively impact hollow-dependent fauna (e.g. birds, bats, mammals and invertebrates), but also will impact a much wider range of plant and animal species that rely on large old trees and associated habitat structures (e.g. coarse woody debris, litter, peeling bark) for a range of purposes (e.g. foraging, spatial connectivity, epiphyte attachment). Ultimately, these species may face local extinction in urban landscapes. This is supported by recent research, which demonstrates that the removal of large old trees from existing urban habitats will likely impact animal populations and community assemblages [19], [20].

Predictions under existing management practices also highlight the important role that nature reserves play in bridging resource gaps across urban landscapes. In contrast to urban greenspace, we predicted that nature reserves adjacent to urban areas provide a relatively stable supply of hollow-bearing trees over time. Therefore, maintaining and establishing nature reserves in urban environments will likely provide important habitat refuge for species over the long term. However, nature reserves only represent a small proportion of the urban landscape and on their own are unlikely to achieve biodiversity conservation targets [58]. In addition, many species rely on networks of multiple habitat trees that extend over large areas of the landscape, including urban habitats [59]. For these reasons, we strongly encourage management strategies that focus on arresting large old tree decline within the ‘working’ urban matrix. This means that a re-evaluation of existing management practices in urban landscapes is needed to address the underlying drivers of tree decline.

### 4.2. Alternative management strategies

Large old trees are especially susceptible to removal in urban landscapes worldwide [20], [28], [29], [60]. With this in mind, we have formulated a set of targeted recommendations, based on results from our analyses, which we anticipate to be relevant to practitioners in a wide range of urban landscapes where trees are maintained.

#### 4.2.1. Maximise tree standing life

A major source of tree mortality in urban landscapes is due to managed tree removal [20]. This is facilitated by public safety policies and practices, which aim to minimise risk of injury to people and damage to property due to falling trees and branches. For example, in our study area it is estimated that by 2050, approximately 175,600 street trees (24% of all trees in urban greenspace) will have reached their safe standing life (ranging from 60 to 100 years old) and are likely to be removed [54]. Consequently, large old trees, hollow-bearing trees, dead trees and decaying branches are most susceptible to targeted removal prior to reaching their full potential in terms of forming and providing suitable habitat. We found that the number of hollow-bearing trees perpetuated in urban greenspace over the long term was most sensitive to the maximum standing life of trees (Table 2; Fig. 4A). Increasing the standing life of all trees by 50 years is predicted to increase the number of hollow-bearing trees ha^−1^ in urban greenspace by 22% over the long term.

Policymakers need to recognize the important habitat resources provided by large old trees and accordingly formulate or amend tree management protocols so that large old trees are afforded better protection. This may involve re-evaluating criteria used to guide tree felling decisions [29]. Practical strategies that maximise the safe standing life of trees should also be implemented. This may involve: (1) allowing trees to age more naturally in urban greenspace frequented less by members of the public and where risk to people and property is minimal (e.g. derelict land, areas along stormwater wetlands, and some parklands); (2) avoiding structural damage to trees (e.g. damage to roots due to road works); (3) creating safe zones or barriers that separate the public from potentially hazardous trees thereby minimising safety risks (e.g. landscaping around the base of the tree using shrubs); (4) physically re-enforcing the structural integrity of large, old trees (e.g. supporting frames, cables or poles); and (5) safely retaining dead trees wherever possible. However, our results indicated that management strategies that only maximise the standing life of trees will be insufficient at mitigating the decline of hollow-bearing trees over the long term (Fig. 6).

#### 4.2.2. Increase tree regeneration

We found that the rate of tree regeneration in urban greenspace (both natural and planted) was 13 times lower than in nature reserves (Fig. 2). A lack of young trees is a major contributing factor of large old tree decline in urban greenspace over the long term. Older trees that eventually die and are removed from any given landscape need to be replaced by younger trees, thereby perpetuating the formation of important habitat structures over multiple generations [18], [61]. We predicted that increasing tree regeneration by 10 native seedlings ha^−1^ would increase the number of hollow-bearing trees in urban greenspace by 10% over the long term (Fig. 4B).

Tree regeneration in urban habitats is typically achieved through planting initiatives and encouraging natural regeneration. Increasing the number of planted trees through government and community initiatives should increase the number of young trees persisting in urban habitats [62]. However, in some urban greenspace (e.g. roadside margins and residential areas), tree planting can be logistically challenging as practitioners need to balance multiple socio-economic and ecological factors when implementing planting strategies, including: site location, public safety, aesthetics, land ownership, and existing vegetation [63]. Furthermore, reducing seedling mortality in urban habitats is also an important consideration that may require additional protection measures (e.g. tree guards, supporting posts) and costs [64]. In some urban greenspace (e.g. parklands, wetlands) it may be more cost-effective over the long term to promote natural regeneration. Natural regeneration in urban habitats is predominantly limited because of: unfavourable seedbed conditions (e.g. impervious surfaces, pollution, and nutrient runoff), increased competition from invasive plants, and increased mortality due to mowing and pedestrian traffic [31], [65]. Strategies that promote natural regeneration could involve: fencing-off areas with existing re-growth, increasing public awareness of regenerating areas through signage, and enhancing local microclimates that favour seedling establishment and survival such as retaining litter and logs [31], [66]. However, our results indicated that management strategies based solely on increasing tree regeneration will be insufficient at mitigating the decline of hollow-bearing trees over the long term (Fig. 6).

#### 4.2.3. Accelerate the formation of habitat structures provided by large old trees

The formation of habitat structures such as hollows is a slow process more likely to occur in large old trees [35]. This is because trees with compromised structural integrity are more susceptible to wood decay resulting in the formation of hollows and other structures such as fallen logs and dead branches. Strategies promoting the formation of habitat structures by artificial means can bypass the time needed for these structures to form naturally. Our results indicate that the density of hollow-bearing trees could be increased in urban greenspace by accelerating hollow formation (Fig. 4C).

Accelerating hollow formation in urban areas is commonly achieved by replicating hollow structures, such as installing artificial nest boxes [32]. However, in urban areas, there are limitations with artificial habitat structures, including: occupancy by pest species, poor rates of target species occupancy, and rapid rates of attrition through collapse and decay of materials [67]. It may also not be feasible or practicable to install and maintain artificial habitat structures in large enough numbers across extensive areas over centuries. Therefore, strategies that accelerate the formation of habitat structures by other means should also be explored [68]. Methods previously proposed for hollows include: tree ringbarking or girdling [69], canopy topping [70], controlled fire burns [71], and injecting trees with herbicides [72]. These strategies are also likely to accelerate the formation of other important habitat structures provided by large old trees, including dead branches and coarse woody debris. In urban landscapes, sub-lethal methods of accelerating habitat structure formation are most preferable to also avoid compromising public safety. This may involve only partially injuring trees by carving out hollows on trunks and some branches [73] and using more invasive methods on trees with large diameters that are structurally robust in order to also maximise tree standing life [35]. More research is still needed to investigate methods aimed at accelerating habitat structure formation, especially in urban landscapes. Nevertheless, our results highlight that management strategies based solely on accelerating hollow formation can be effective at increasing the density of hollow-bearing trees in the short-term, but not over the long term (Fig. 6).

#### 4.2.4. Our management proposal: A combined management approach is needed

Our results emphasise that a combination of different management approaches, aimed at improving multiple aspects of tree management and maintenance, are needed to perpetuate hollow-bearing trees in urban greenspace over the long term (Fig. 5). We propose a management strategy based on simultaneously manipulating all three explanatory variables discussed above, which were identified as being the most sensitive model parameters in our analyses. Under this scenario (Fig. 6), we predicted that the decline of hollow-bearing trees in urban greenspace can be arrested within 250 years if: (1) trees remain standing for at least 450 years ensuring that they reach their maximum habitat potential; (2) at least 60 seedlings ha^−1^ are planted or naturally regenerated; and (3) hollow formation is accelerated to a rate of 2.0 in the short term by installing nest boxes and sub-lethally creating hollows by other methods. Our proposal considers the complexities associated with managing urban greenspaces for multiple purposes, including recreation and conservation. We recognize that it may not be possible to retain all trees to their maximum biological age due to public safety risks. It may also not be practical or feasible to accelerate the formation of habitat structures artificially on a large enough scale over prolonged time periods. Instead, we attempt to balance socio-economic and biodiversity benefits by combining multiple tree management and maintenance approaches in an achievable manner. Future research should also aim to investigate alternative management scenarios from a more financial perspective, which too would benefit practitioners (e.g. numbers of hollow-bearing trees gained per management dollar spent). However, even under our proposed management strategy, the density of hollow-bearing trees is predicted to first decline, or undergo a bottleneck, before increasing. This is because of an extinction debt or the time lag between implementing management actions and actually observing an increase in hollow-bearing trees. Delaying mitigation is anticipated to further exacerbate the effects of time lags and require more drastic measures at greater costs to reverse tree declines [26]. Immediate action will likely also reduce bottlenecks in urban plant and animal populations that depend on large old trees for survival.

## Conclusion

We have quantified the decline of hollow-bearing trees in an urban landscape over centuries. We provided a novel assessment of the conservation implications associated with existing tree management practices and the efficacy of a range of alternative management strategies. It is evident from our results that existing urban tree management practices require urgent re-evaluation if hollow and tree-dependent biodiversity are to be maintained in urban landscapes. We recommend that: (1) large old trees are afforded better protection and remain standing over longer time periods; (2) tree regeneration is actively improved so that large old trees lost over time are replaced by younger trees; and (3) the formation of habitat structures provided by large old trees is accelerated to compensate for short term deficits in resource availability. Immediate implementation of these recommendations is needed to arrest the decline of large old trees, avoid lag effects, and avert long term risk to biodiversity in urban landscapes.

## Supporting Information

Table S1 **List of recorded tree species and diameter size class distributions.**
(DOCX)

Summary S1 **Description of the simulation model used for analyses.**
(DOCX)
