# Supplementary material for: The Future of Large Old Trees in Urban Landscapes
Source: PLoS One. 2014 Jun 18;9(6):e99403. doi: 10.1371/journal.pone.0099403 (PMC4062419; doi:10.1371/journal.pone.0099403)
Supplement: Table S1 — List of recorded tree species and diameter size class distributions. (DOCX) [file pone.0099403.s001.docx]

**Table S1.** List of recorded tree species and diameter size class distributions

| **A. Nature reserves** | **Tree species** | **Diameter size class (cm)** | | | | | | | | | | |
| --- | --- | --- | --- | --- | --- | --- | --- | --- | --- | --- | --- | --- |
|  |  | 0.1-10 | 11-20 | 21-30 | 31-40 | 41-50 | 51-60 | 61-70 | 71-80 | 81-90 | 91-100 | >100 |
|  | *E. blakelyi* (Blakely’s red gum) | 1,595 | 122 | 39 | 27 | 3 | 16 | 9 | 4 | 5 | 8 | 9 |
|  | *E. bridgesiana* (apple box) | 244 | 58 | 49 | 9 | 1 | 6 | 2 | 2 | 3 | 0 | 2 |
|  | *E. dalrympleana* (mountain gum) | 1 | 0 | 0 | 0 | 0 | 0 | 0 | 1 | 0 | 0 | 0 |
|  | *E. dives* (broad-leaved peppermint) | 10 | 0 | 0 | 1 | 1 | 0 | 1 | 0 | 0 | 0 | 0 |
|  | *E. goniocalyx* (bundy) | 0 | 0 | 1 | 0 | 0 | 0 | 0 | 0 | 0 | 0 | 0 |
|  | *E. macrorhyncha* (red stringybark) | 60 | 39 | 17 | 7 | 2 | 0 | 2 | 0 | 0 | 0 | 1 |
|  | *E. mannifera* (brittle gum) | 5 | 0 | 0 | 1 | 1 | 0 | 1 | 0 | 0 | 0 | 0 |
|  | *E. melliodora* (yellow box) | 948 | 153 | 57 | 20 | 5 | 13 | 3 | 8 | 9 | 2 | 12 |
|  | *E. nortonii* (mealy bundy) | 2 | 0 | 0 | 0 | 0 | 0 | 0 | 0 | 0 | 0 | 0 |
|  | *E. polyanthemos* (red box) | 69 | 34 | 9 | 2 | 0 | 0 | 1 | 2 | 0 | 0 | 0 |
|  | *E. rossii* (scribbly gum) | 216 | 32 | 21 | 10 | 1 | 4 | 2 | 0 | 0 | 1 | 1 |
|  | Dead trees | 38 | 43 | 9 | 7 | 3 | 4 | 3 | 2 | 1 | 1 | 0 |
| **Sum** |  | **3,188** | **481** | **202** | **84** | **17** | **43** | **24** | **19** | **18** | **12** | **25** |
| **B. Urban greenspace** |  |  |  |  |  |  |  |  |  |  |  |  |
|  | *E. blakelyi* (Blakely’s red gum) | 90 | 22 | 29 | 23 | 11 | 5 | 4 | 6 | 2 | 3 | 5 |
|  | *E. bridgesiana* (apple box) | 14 | 4 | 12 | 3 | 0 | 1 | 0 | 0 | 0 | 0 | 0 |
|  | *E. dalrympleana* (mountain gum) | 1 | 0 | 0 | 1 | 2 | 0 | 2 | 1 | 3 | 0 | 0 |
|  | *E. delegatensis* (alpine ash) | 0 | 0 | 0 | 0 | 1 | 0 | 0 | 0 | 0 | 0 | 0 |
|  | *E. dives* (broad-leaved peppermint) | 1 | 1 | 2 | 5 | 4 | 3 | 1 | 1 | 2 | 0 | 0 |
|  | *E. fastigata* (brown barrel) | 0 | 0 | 3 | 0 | 2 | 0 | 0 | 0 | 0 | 0 | 0 |
|  | *E. goniocalyx* (bundy) | 1 | 2 | 0 | 2 | 0 | 0 | 0 | 0 | 0 | 0 | 0 |
|  | *E. macrorhyncha* (red stringybark) | 0 | 1 | 0 | 2 | 4 | 3 | 3 | 1 | 0 | 1 | 1 |
|  | *E. mannifera* (brittle gum) | 20 | 21 | 37 | 26 | 11 | 7 | 3 | 2 | 1 | 0 | 0 |
|  | *E. melliodora* (yellow box) | 68 | 26 | 29 | 10 | 11 | 10 | 3 | 4 | 1 | 4 | 6 |
|  | *E. nortonii* (mealy bundy) | 2 | 1 | 1 | 1 | 1 | 0 | 0 | 0 | 0 | 0 | 0 |
|  | *E. polyanthemos* (red box) | 1 | 8 | 5 | 3 | 1 | 0 | 1 | 0 | 0 | 0 | 0 |
|  | *E. rossii* (scribbly gum) | 38 | 6 | 4 | 1 | 0 | 0 | 0 | 1 | 0 | 0 | 0 |
|  | *E. rubida* (candlebark) | 8 | 1 | 2 | 4 | 4 | 0 | 0 | 1 | 0 | 0 | 0 |
|  | *E. sideroxylon* (ironbark) | 1 | 5 | 7 | 2 | 2 | 3 | 4 | 1 | 0 | 0 | 0 |
|  | *E. viminalis* (ribbon gum) | 4 | 3 | 8 | 9 | 5 | 3 | 2 | 2 | 0 | 1 | 1 |
|  | Dead trees | 1 | 1 | 0 | 3 | 0 | 0 | 0 | 0 | 0 | 0 | 0 |
| **Sum** |  | **249** | **102** | **139** | **95** | **62** | **35** | **23** | **20** | **9** | **9** | **13** |
