# Supplementary material for: The Future of Large Old Trees in Urban Landscapes
Source: PLoS One. 2014 Jun 18;9(6):e99403. doi: 10.1371/journal.pone.0099403 (PMC4062419; doi:10.1371/journal.pone.0099403)
Supplement: Summary S1 — Description of the simulation model used for analyses. (DOCX) [file pone.0099403.s002.docx]

**Summary S1.** Description of the simulation model used for analyses

The simulation model we used in our paper to predict the size-class distribution of trees in stands over time was developed using Visual Basic in Microsoft Excel and can be obtained from the authors on request. This simulation model is also described with respect to the management of scattered trees in agricultural landscapes [1], mature trees in wood production forests [2], and nest trees for a threatened species in [3].

This simulation model tracks the mean diameter at breast height (DBH) of trees in cohorts through time. For this exercise, we separately simulated trees in reserves and urban areas. We employed a Monte Carlo approach to simulation, whereby parameters in the model can be entered as random values within a range of observed or likely values so the predictions reflect the uncertainty of these parameters. The model is then run as many times as is specified (in this instance all predictions were based on 300 runs of the model), so predictions for any single scenario are a summary of values from multiple runs of the model. There are six key steps in the simulation.

**Step 1 – Record the numbers of trees by size-class**

The simulation commences with data on the mean number of trees in 10 cm diameter classes (i.e., 0-10 cm, 11-20 cm, etc.) for each tree species group (species distributions summarised in Table S1). Initial values at the commencement of the simulation (*T*=0 years) were based on data collected at 200 50 m x 20 m (0.1 ha) plots in urban and nature reserve sites within our study area. These data are expressed as a per ha basis for each simulation.

**Step 2 – Estimate the numbers of trees with hollows**

We predicted the proportion of trees that contained hollows separately for each tree species group and DBH cohort based on visual estimation of the presence/absence of hollows in trees across the study area using the equation: *Logit (Pr. Hollows)* = -7.112 + (0.086 x DBH) + (species group estimate). The area under the receiver operating characteristic curve of this statistical model was 0.92, indicating that its discriminating ability was excellent [4].

**Step 3 – Grow trees for *t* years**

To simulate tree-growth over time, we developed a relationship between tree age and DBH using the following equation developed by [5]: Age = 0.02 × π × (DBH_standardized_ /2)^2^, where DBH_standardised_ is the yellow box (*Eucalyptus melliodora*) equivalent diameter for each tree as defined below. Data on the relationship between age and DBH only exist for one tree species (yellow box) in our study area [6]. To predict the ages of trees of other species, we followed the procedure outlined by [5] and calculated a yellow box equivalent diameter for each individual tree of the other eucalypt species. The procedure assumed that all eucalypt species in our study area follow an identical growth curve relative to their maximum attainable diameter, and have the same approximate life-span as yellow box. DBH values for all tree species were initially standardised as a proportion of the maximum attainable diameter for that species observed in the field. Those values were then multiplied by the maximum diameter observed for yellow box (151 cm) to obtain a yellow box equivalent diameter. We acknowledge that this procedure is unlikely to give precise age estimates, but it is a pragmatic solution given the paucity of data on tree ages available for trees in our study area. However, previous research [1,2] indicates that the number of mature trees perpetuated over time is not sensitive to this variable. The initial age of trees in each cohort is predicted by using the median DBH of each cohort in Equation 2, *t* years is added to this age (where *t* is the years between recruitment events) and then the inverse of Equation 2 is used, in turn, to predict the new DBH of the cohort after *t* years.

**Step 4 – Recruit a specified number of trees at the beginning of each time-step**

We recruited *n* new trees per ha into each landscape type (urban and reserve) every *t* years. For the *status quo* scenarios, *n* was the mean number of trees by tree species group in the smallest DBH class within each landscape and *t* was the age of this cohort (estimated using Equation 2). Recognising that tree recruitment can be highly variable from year to year, the value for *n* for each tree species group in each landscape type for the *status quo* scenarios was a random value drawn from a Poisson distribution with a mean taken from the smallest diameter cohort (0-10 cm DBH) for trees in each species group within each landscape type. For the alternative management scenarios in urban areas, we chose random values from a uniform distribution. Runs with negative values for recruitment were treated as zero recruitment.

**Step 5 – apply tree mortality**

We applied two sources of mortality during each time-step. We calculated tree mortality from data collected on changes in the mean numbers of trees in each DBH cohort, reflecting the density-dependent nature of tree mortality in natural stands. This was given as: 1 – *s* ^(1 /y)^, where *s* is the proportion of trees that survive from one cohort to the next, and *y* is the number of years between recruitment events.

For trees in reserves, we set 500 as the maximum number of years that trees will remain living, which is based on longevity estimates for yellow box reported by [6]. There were no other data from which this estimate could be derived and [1] reports that the number of scattered trees is not sensitive to this parameter in simulations of this type. For urban areas, the maximum number of years that trees remain living was selected randomly from a uniform distribution between 60-500 years, reflecting the existing policy of the government in our study area to remove trees as young as 60 years old for safety reasons. Once a tree had died, we allowed it to remain standing for 50 years in reserves, but in urban areas we removed the tree immediately in keeping with management practices in our study area.

At the completion of this step the number of surviving trees by DBH class and species group was tallied.

The predicted proportion of hollow-bearing tree was then multiplied by the mean numbers of surviving trees in each DBH cohort to arrive at a predicted number of trees with hollows at the end of each time-step (*t*).

Steps 1-5 are repeated such that *t* (the period between regeneration events) is added to *T* (the total length of the simulation) until *T*=300 years*.* We reported the mean (±95% prediction interval) for all runs of the simulation model at each time-step between 0 and 300 years.

**References**

1. Gibbons P, Lindenmayer DB, Fischer J (2008) The future of scattered trees in agricultural landscapes. Conservation Biology 22: 1309-1319.

2. Gibbons P, McElhinny C, Lindenmayer DB (2010) What strategies are effective for perpetuating structures provided by old trees in harvested forests? A case study on trees with hollows in south-eastern Australia. Forest Ecology and Management 260: 975-982.

3. Manning AD, Gibbons P, Fischer J, Oliver DL, Lindenmayer DB (2012) Hollow futures? Tree decline, lag effects and hollow-dependant species. Animal Conservation 16: 395-405.

4. Pearce J, Ferrier S (2000) Evaluating the predictive performance of habitat models developed using logistic regression. Ecological Modelling 133: 225-245.

5. Fischer J, Zerger A, Gibbons P, Stott J, Law B (2010) Tree decline and the future of Australian farmland biodiversity. PNAS 107: 19597-19602.

6. Banks JCG (1997) Tree ages and ageing in yellow box. In: Dargavel J, editor. The coming of age Forest age and heritage values. Canberra, Australia: Environment Australia. pp. 17-28.
